# Mutations in *Caenorhabditis elegans *actin, which are equivalent to human cardiomyopathy mutations, cause abnormal actin aggregation in nematode striated muscle

**DOI:** 10.12688/f1000research.18476.1

**Published:** 2019-03-12

**Authors:** Yuriko Hayashi, Kanako Ono, Shoichiro Ono

**Affiliations:** 1 Department of Pathology, Emory University, Atlanta, Georgia, 30322, USA; 2 Department of Cell Biology, Emory University, Atlanta, Georgia, 30322, USA; 3 Winship Cancer Institute, Emory University, Atlanta, Georgia, 30322, USA

**Keywords:** actin, aggregates, cardiomyopathy, sarcomere, myofibrils

## Abstract

Actin is a central component of muscle contractile apparatuses, and a number of actin mutations cause diseases in skeletal, cardiac, and smooth muscles. However, many pathogenic actin mutations have not been characterized at cell biological and physiological levels. In this study, we tested whether the nematode *Caenorhabditis elegans *could be used to characterize properties of actin mutants in muscle cells *in vivo*. Two representative actin mutations, E99K and P164A, which cause hypertrophic cardiomyopathy in humans, are introduced in a muscle-specific *C. elegans *actin ACT-4 as E100K and P165A, respectively. When green fluorescent protein-tagged wild-type ACT-4 (GFP-ACT-4), is transgenically expressed in muscle at low levels as compared with endogenous actin, it is incorporated into sarcomeres without disturbing normal structures. GFP-ACT-4 variants with E100K and P165A are incorporated into sarcomeres, but also accumulated in abnormal aggregates, which have not been reported for equivalent actin mutations in previous studies. Muscle contractility, as determined by worm motility, is not apparently affected by expression of ACT-4 mutants. Our results suggest that *C. elegans *muscle is a useful model system to characterize abnormalities caused by actin mutations.

## Introduction

Actin is an essential component of the cytoskeleton in both muscle and non-muscle cells. A number of mutations in the six human actin genes cause a wide range of diseases in various tissues ( Despond & Dawson, 2018; North & Laing, 2008; Rubenstein & Wen, 2014). In muscles, actin, together with myosin, generates contractile forces, and therefore, alterations in contractile and/or structural properties of actin can cause muscle malfunction. Mutations in skeletal muscle α-actin ( *ACTA1*) cause congenital myopathies, including nemaline myopathy and intranuclear rod myopathy, in which skeletal muscle exhibits abnormal accumulations of sarcomeric components ( Clarkson *et al*., 2004; Laing *et al*., 2009; North & Laing, 2008; Ono, 2010). Many of these cytoskeletal abnormalities can be reproduced by expression of mutant actins in cultured non-muscle or muscle cells ( Bathe *et al*., 2007; Costa *et al*., 2004; Domazetovska *et al*., 2007; Vandamme *et al*., 2009a; Vandamme *et al*., 2009b) or in transgenic mice ( Lindqvist *et al*., 2013; Ravenscroft *et al*., 2011). By contrast, mutations in cardiac α-actin ( *ACTC1*) cause hypertrophic and dilated cardiomyopathies ( Mogensen *et al*., 1999; Olson *et al*., 1998). Biochemical studies indicate that these cardiomyopathy mutations of actin alter its properties to generate contractile forces ( Despond & Dawson, 2018). However, abnormalities in sarcomeric or cytoskeletal structures have not been reported when the mutant actins are expressed in cultured cells ( Muller *et al*., 2012; Vang *et al*., 2005) or transgenic mice ( Song *et al*., 2010; Song *et al*., 2011).

In this study, we used the nematode *Caenorhabditis elegans* as a model to examine effects of cardiomyopathy mutations in actin. The body wall muscle of *C. elegans* is obliquely striated muscle with a number of functional and structural similarities to vertebrate striated muscles ( Ono, 2014). Four actin genes are expressed in *C. elegans* muscle ( Files *et al*., 1983; Stone & Shaw, 1993), and they are 95% identical to human cardiac and skeletal muscle α-actins ( Ono & Pruyne, 2012). Since all known residues that are mutated in human cardiomyopathies are conserved in *C. elegans* actins, we selected two representative hypertrophic cardiomyopathy mutations and tested whether these pathogenic mutations perturb the properties of actin in *C. elegans* muscle *in vivo*. We found that the mutant actins were incorporated into sarcomeres and also accumulated in abnormal aggregates, suggesting that *C. elegans* muscle is a unique model system to characterize pathogenic actin mutations.

## Methods

### Worm culture

Worms were cultured following standard methods ( Stiernagle, 2006). Wild-type *C. elegans* strain N2 was obtained from the *Caenorhabditis* Genetics Center (Minneapolis, MN) and used in this study.

### Transgenic strains

An expression vector for GFP-ACT-4(wild-type: WT) was constructed by inserting ACT-4 cDNA at the *Eco*RI- *Nhe*I sites of pPD118.20 (provided by Andrew Fire, Stanford University) in-frame with the 3’-end of the GFP coding sequence. Briefly, first-strand cDNAs were reverse-transcribed from total RNAs from the N2 strain using oligo-dT by a Maxima H ^-^ First Strand cDNA Synthesis Kit (Thermo Fisher Scientific). The ACT-4 cDNA with added *Eco*RI and *Nhe*I sites in the primer sequences was amplified from the pool of cDNAs by polymerase chain reaction using *Pfu* DNA polymerase (Agilent Technologies), digested with *Eco*RI and *Nhe*I, and ligated with pPD118.20 that had been cut with *Eco*RI and *Nhe*I. Expression vectors for GFP-ACT-4(E100K) and GFP-ACT-4(P165A) were generated by site-directed mutagenesis using a QuickChange Site-directed Mutagenesis Kit (Agilenet Technologies). Sequences of the inserts were verified by DNA sequencing. Transgenic nematodes were generated by microinjection of DNA vectors into the distal gonads as described previously ( Mohri *et al*., 2006). Transgenic worms were selected by expression of GFP as observed by fluorescence microscopy, and the transgenes were maintained as extrachromosomal arrays. Strains used in this study are ON16, *ktEx6[Pmyo-3::GFP::ACT-4(WT)]*; ON209, *ktEx154[Pmyo-3::GFP::ACT-4(E100K)]*; and ON212, *ktEx157[Pmyo-3::GFP::ACT-4(P165A)]*.

### Western blot

Ten adult worms were suspended in 15 µl SDS lysis buffer (2% SDS, 80 mM Tris-HCl, 5% β-mercaptoethanol, 15% glycerol, 0.05% bromophenol blue, pH 6.8), heated at 97°C for 2 min, homogenized briefly by sonication, heated again at 97°C for 2 min, and subjected to SDS-PAGE (12% acrylamide gel). The proteins were transferred to a polyvinylidene difluoride membrane (Immobilon-P, Millipore). The membrane was blocked in 5% nonfat milk in phosphate-buffered saline (PBS) containing 0.1% Tween 20 (PBS-T) and incubated for 1 hr with anti-actin mouse monoclonal antibody (C4, MB Biomedicals, catalog # 08691001; RRID:AB_2335127) at a 1:3000 dilution. The membrane was washed with PBS-T, treated with horseradish peroxidase-conjugated goat anti-mouse IgG (1:2000 dilution) (Pierce/Thermo Scientific, catalog #31430) for 1 hr, and washed with PBS-T. The reactivity was detected with SuperSignal West Pico Chemiluminescent Substrate (Thermo Scientific) and exposure to X-ray films. Finally, the membrane was stained with 0.1% Coomassie Brilliant Blue R-250 (National Diagnostics) in 50% methanol and destained in a solution containing 10% acetic acid and 50% methanol to visualize total proteins ( Welinder & Ekblad, 2011). The blots were scanned by an Epson Perfection V700 scanner at 300 dpi., and band intensity was quantified using ImageJ 1.47v.

### Worm motility assay

Worm motility was determined by counting swinging motions of worms for 30 seconds in M9 buffer as described ( Epstein & Thomson, 1974; Ono *et al*., 1999).

### Fluorescence microscopy

Fixation and staining of worms with rhodamine-phalloidin were performed as described previously ( Ono, 2001). GFP was observed by its own fluorescence. Specimens were observed by epifluorescence using a Nikon Eclipse TE2000 inverted microscope with a CFI Plan Fluor ELWD 40x (Dry; NA 0.60) objective. Images were captured by a SPOT RT monochrome CCD camera (Diagnostic Instruments) and processed by IPLab 4.0 imaging software (BD Biosciences) and Adobe Photoshop CS3.

### Molecular graphics

**Figure 1.**
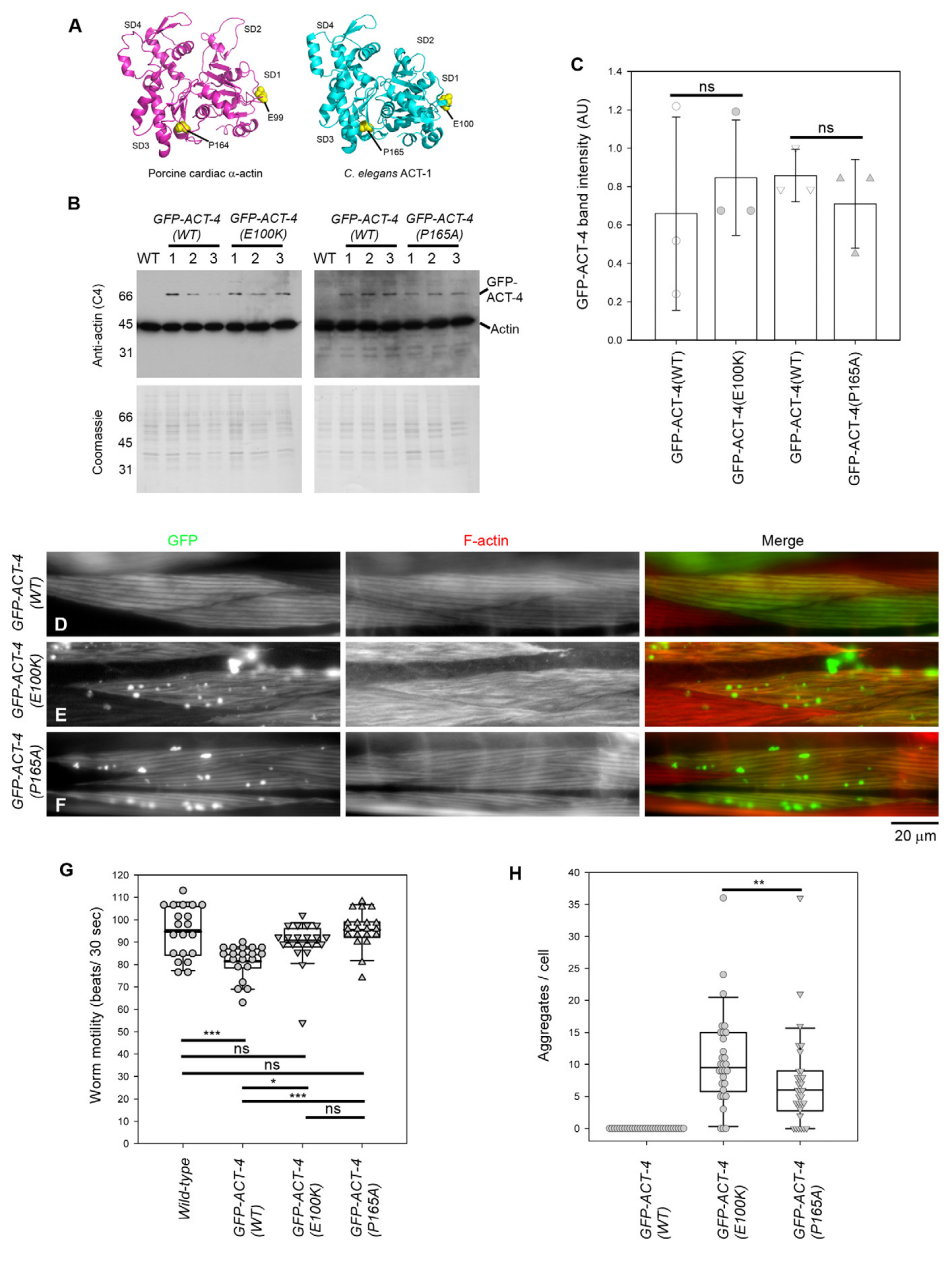
Effects of expression of GFP-ACT-4 variants in *C. elegans* body wall muscle. (A) Structure of porcine cardiac α-actin ( Risi *et al*., 2017) (Protein Data Bank accession number 5N0J) and *C. elegans* ACT-1 ( Vorobiev *et al*., 2003) (Protein Data Bank accession number 1D4X). ACT-1 is also expressed in *C. elegans* muscle and differs from ACT-4 by only one amino acid. Mutated residues (E99 and P164 in porcine cardiac α-actin; E100 and P165 in *C. elegans* ACT-1) are shown in yellow. Actin subdomains 1-4 are labeled as SD1-SD4. Molecular graphics were generated by PyMol (Schrödinger). (B) Western blot analysis of expression levels of GFP-ACT-4 variants. Total worm lysates (10 worms each) from wild-type without a transgene (WT) or with transgenes expressing GFP-ACT-4 variants were analyzed by western blot using an anti-actin antibody (top). Coomassie Brilliant Blue staining of the membranes after chemiluminescence detection (bottom) was used to normalize protein loading. Positions of GFP-ACT-4 (70 kDa) and endogenous actin (42 kDa) are indicated on the right. Representative molecular weight markers in kDa are indicated on the left. For each transgenic strain, three independently prepared lysates (#1-3) were analyzed. (C) Quantitative analysis of the Western blot (Dataset 1). Band intensity in arbitrary units (AU) of GFP-ACT-4 was normalized to intensity of total protein staining by Coomassie Brilliant Blue ( Welinder & Ekblad, 2011) and plotted on the graph. GFP-ACT-4(WT) and each GFP-ACT-4 mutant were compared on the same western blot, and no significant differences were found by Student’s t-test (ns) (n=3). (D-F) Localization patterns of GFP-ACT-4 (left) and F-actin (middle) in the *C. elegans* body wall muscle from worms expressing GFP-ACT-4(WT) (D), GFP-ACT-4(E100K) (E), and GFP-ACT-4(P165A) (F). Merged images (GFP in green and F-actin in red) are shown on the right. Bar, 20 µm. (G) Worm motility of each strain was examined by beating frequency (beats per 30 sec) (Dataset 2). The results were analyzed by one-way ANOVA (n=20): ns, not significant (p>0.05); *p<0.05 p<0.01; **p<0.01; and ***p<0.001. (H) Number of GFP-ACT-4 aggregates per cell was counted (Dataset 3). The results were analyzed by one-way ANOVA (n=30) and significant difference was found between the data for GFP-ACT-4(E100K) and GFP-ACT-4(P165A) (**p = 0.006).

Molecular graphics in Figure 1A were generated using PyMol 2.1.0 (Schrödinger), and texts added using Adobe Photoshop CS3.

### Statistical analysis

The data used in Figure 1C were analyzed by Student’s t-test using SigmaPlot 14.0 (Systat Software, Inc.). The data used in Figure 1G were analyzed by one-way ANOVA with Turkey test using SigmaPlot 14.0. The data used in Figure 1H were analyzed by one-way ANOVA with pairwise multiple comparison using the Student-Newman-Keuls method using SigmaPlot 14.0.

## Results

We constructed an expression vector for GFP-tagged ACT-4, an actin isoform that is expressed in the body wall muscle ( Stone & Shaw, 1993), under the control of the *myo-3* promotor ( *Pmyo-3*) ( Okkema *et al*., 1993). The ACT-4 sequence was fused to the C-terminus of GFP with a 9-residue linker sequence (SPQALEFSS) to minimize the interference of actin function by GFP ( Aizawa *et al*., 1997). We selected two missense mutations, E99K and P164A in human cardiac α-actin ( Despond & Dawson, 2018; Olson *et al*., 2000), that dominantly cause hypertrophic cardiomyopathy. The E99K mutation weakens actin-myosin interaction ( Bookwalter & Trybus, 2006) and increases the critical concentration of actin ( Mundia *et al*., 2012). In a transgenic mouse model, E99K increases calcium sensitivity of the thin filaments and causes abnormal heart functions ( Song *et al*., 2011). In contrast, the effect of P164A mutation remains unclear. Although P164A causes alteration in protein folding *in vitro* ( Vang *et al*., 2005), an equivalent mutation in yeast actin does not change its basic biochemical properties ( Wong *et al*., 2001). *C. elegans* ACT-4 is 95% identical in amino acid sequence to human cardiac α-actin, and E99 and P164 are conserved as E100 and P165, respectively ( Figure 1A). Therefore, we introduced E100K and P165A mutations in GFP-ACT-4 and examined their effects on the sarcomeric structures in *C. elegans* body wall muscle.

### Establishment of transgenic strains and expression quantification

We established at least three independent transgenic strains for each of the transgenes, GFP-ACT-4(wild-type: WT), GFP-ACT-4(E100K), and GFP-ACT-4(P165A), and examined expression levels of GFP-ACT-4 variants by western blot. We selected one strain each, which expressed the GFP-ACT-4 variants at similar levels ( Figure 1B, C) for further analysis. Western blot analysis using anti-actin antibody showed that all the GFP-ACT-4 variants were expressed at much lower levels than endogenous actin in total worm lysates ( Figure 1B). The level of GFP-ACT-4(WT) was roughly estimated by densitometry to be lower than 10% of that of total endogenous actin, although strong saturated signals for endogenous actin made precise quantification difficult. Considering that body wall muscle is the major tissue expressing actin as a sarcomeric component, the expression level of GFP-ACT-4 should be still much less than that of endogenous actin within the body wall muscle cells. Raw uncropped western blots, alongside all other raw data, are available on Figshare ( Hayashi *et al*., 2019).

### Subcellular localization of GFP-ACT-4 variants and motility of worms

GFP-ACT-4(WT) was incorporated into sarcomeres in body wall muscle cells ( Figure 1D). Staining of F-actin in fixed animals with rhodamine-phalloidin showed a nearly identical localization pattern to GFP-ACT-4(WT) ( Figure 1D). Motility of the worms expressing GFP-ACT-4(WT) (81.5 ± 7.5 beats/30 sec, n = 20), as determined by beating frequency in liquid, was slightly slower than that of wild-type worms with no transgene (94.8 ± 11 beats/30 sec, n = 20), suggesting that GFP-ACT-4(WT) has a weak negative effect on contractility of the body wall muscle.

Both GFP-ACT-4(E100K) and GFP-ACT-4(P165A) were incorporated into sarcomeres but also formed spherical aggregates in the cytoplasm of the body wall muscle cells ( Figure 1E, F). Staining with rhodamine-phalloidin showed that sarcomeric organization of actin filaments were somewhat disorganized by expression of GFP-ACT-4(E100K) ( Figure 1E) but not GFP-ACT-4(P165A) ( Figure 1F). However, motility of the worms expressing GFP-ACT-4(E100K) or GFP-ACT-4(P165A) was not significantly different from that of wild-type worms ( Figure 1G), suggesting that these actin mutants did not disturb muscle contractility. These aggregates resemble F-actin aggresomes induced by inhibitors of actin dynamics ( Lázaro-Diéguez *et al*., 2008). However, the aggregates of GFP-ACT-4(E100K) or GFP-ACT-4(P165A) were not recognized by rhodamine-phalloidin, a specific probe for F-actin ( Figure 1E, F). In addition, we could not detect these aggregates by immunofluorescence using anti-actin monoclonal or polyclonal antibodies, even after attempts to expose antigens using guanidine hydrochloride ( Peränen *et al*., 1993) or microwave ( Shi *et al*., 1991), suggesting that the mutant forms of actin were present in an inclusion-body-like state and not readily accessible to the actin probes. Such aggregates were not detected in worms expressing GFP-ACT-4(WT) ( Figure 1D, H), while variable numbers (0 - 36 per cell) of aggregates were found in worms expressing GFP-ACT-4(E100K) or GFP-ACT-4(P165A) ( Figure 1E, F). In randomly selected worms (n = 30), GFP-ACT-4(E100K) (median = 9.5 aggregates per cell) induced significantly more aggregates than GFP-ACT-4(P165A) (median = 6.0 aggregates per cell) ( Figure 1H). These aggregates were randomly located in the cytoplasm but not within the nucleus. Thus, we conclude that the missense mutations in ACT-4 induced the formation of abnormal cytoplasmic aggregates in muscle cells.

## Discussion

Formation of actin aggregates by E99K (E100K in worm) or P164A (P165A in worm) mutation in actin has not been reported in human patients or other experimental systems. When cardiac α-actin mutants (E99K and P164A) are expressed in COS-7 cells, these actin mutants are not incorporated in the non-muscle actin cytoskeleton with no detectable aggregate formation ( Vang *et al*., 2005). When E99K cardiac α-actin is expressed in the mouse heart, the mutant actin is incorporated in the cardiac thin filaments and causes disarray of cardiomyocytes but with no detectable aggregate formation ( Song *et al*., 2011). Thus, effects of these actin mutations appear to be dependent on cellular contexts. Formation of actin aggregates by these actin mutations might be specific to the nematode muscle. We also cannot exclude the possibility that the aggregate formation is artificially enhanced by the GFP tag. Nonetheless, we were able to detect actin aggregates because of the GFP tag and might not have been able to detect the aggregates if a fluorescent tag was absent. Abnormal protein aggregates have been reported in idiopathic dilated cardiomyopathy ( Gianni *et al*., 2010; Subramanian *et al*., 2015) and cardiomyopathies caused by mutations in desmin ( McLendon & Robbins, 2011; Sanbe *et al*., 2004), filamin ( Brodehl *et al*., 2016; Reinstein *et al*., 2016; Valdes-Mas *et al*., 2014), α-B-crystallin ( Vicart *et al*., 1998), or phospholamban ( Te Rijdt *et al*., 2016). Whether transient or stable protein aggregates are formed in actin-linked cardiomyopathies remains to be investigated. Our observations suggest that the *C. elegans* might be a relevant model system to study certain types of cardiomyopathies.

## Data availability

Figshare: Raw data - Mutations in Caenorhabditis elegans actin, which are equivalent to human cardiomyopathy mutations, cause abnormal aggregation in nematode striated muscle. https://doi.org/10.6084/m9.figshare.c.4424546 ( Hayashi *et al*., 2019).

This collection contains the following underlying data:

- Uncropped western blots
- Unprocessed microscopy images
- Dataset 1–3 (containing western blot quantification, and raw data for worm motility and number of aggregates per cell)

Data are available under the terms of the Creative Commons Zero "No rights reserved" data waiver (CC0 1.0 Public domain dedication).
