# Supplementary material for: Mutations in Caenorhabditis elegans actin, which are equivalent to human cardiomyopathy mutations, cause abnormal actin aggregation in nematode striated muscle
Source: F1000Res. 2019 Mar 12;8:279. [Version 1] doi: 10.12688/f1000research.18476.1 (PMC6446495; doi:10.12688/f1000research.18476.1)
Supplement: Peer review history

# Peer review: Reviewer response for version 1

**DOI:** 10.5256/f1000research.20214.r45622

**Published:** 2019-04-02

**Authors:** Christophe Ampe

**Affiliations:** 1 Department of Biomolecular Medicine, Faculty of Medicine and Health Sciences, Ghent University, Ghent, Belgium

This reports describe the expression of two mutant actins, associated with cardiac myopathy, in the body wall muscle of the model organism *Caenorhabditis elegans*. Expression of these mutants as N-terminally tagged GFP-variants at low to moderate expression levels (relative to the endogenous actin) disturbs the fine structure of body wall muscle and dot-like patterns of GFP-mutant protein are evident in contrast to the experiments with GFP-WT-ACT-4 (see also below).

 On the whole the experiments are well conducted and properly documented. I have some suggestions regarding interpretation of the results which the authors should look into but this does not require further experimentation (i.e. only textual revision).

 Expression of WT GFP-ACT4 results in a negative effect on worm motility. This is mentioned by the authors in the results (Subcellular localization of GFP-ACT-4 variants and motility of worms). Such an observation is not unprecedented in mammalian cells and is basically due to the GFP—moiety possibly interfering with cellular functions. This was further investigated in Agbulut *et al.* (2007 ^1^) and it was shown that GFP on its own interferes with myosin-based contractility which is potentially also the case here. In this respect should the authors not interpret the worm motility relative to the GFP-ACT-4 control rather than relative to WT worms? I realise it is more difficult to reconcile disturbed muscle structure (D-F) and the expected dominant-negative phenotype with increased worm motility (G) but the authors indicate the motility of the worms is significantly different between mutant and WT-GFP actin variants and this should be commented on in the results or the discussion section.

 In the discussion the authors interpret their results solely in terms of cardiomyopathy based on the origin of the mutations. However the experimental system used is body wall muscle and interestingly the cellular phenotype is more reminiscent of nemaline myopathy (dotted patterns and irregular fibers). Functional differences between alpha-cardiac actin and alpha-skeletal muscle actin in human are unclear because the proteins are highly similar (only 4 amino acids different and these changes are even conservative) and differential phenotypes of disease mutants likely result from differential expression in the respective tissues (in agreement with the authors’ statement: “Thus, effects of these actin mutations appear to be dependent on cellular contexts”). These ACTC1 mutants do not cause aggregates in COS cells (Vang *et al.*, 2005, mentioned in the discussion), therefore I suggest to also make the parallel with nemaline myopathy where dot-like patterns in cellular context of (fused) myoblasts are frequently documented.

 The dots appear to be phalloidin-negative as judged by visual inspection (Figure 1D and 1E); perhaps this should be mentioned in the main text.

**Other minor points:**

Introduction:

- In the two sentences: “Many of these cytoskeletal abnormalities can be reproduced by expression of mutant actins in cultured non-muscle or muscle cells (Bathe *et al*., 2007; Costa *et al*., 2004; Domazetovska *et al*., 2007; Vandamme *et al*., 2009a; Vandamme *et al*., 2009b) or in transgenic mice (Lindqvist *et al*., 2013; Ravenscroft *et al*., 2011). By contrast, mutations in cardiac α-actin ( *ACTC1*) cause hypertrophic and dilated cardiomyopathies (Mogensen *et al*., 1999; Olson *et al*., 1998).” a contrast is mentioned although the two items do not contrast each other.

Methods:

- Correct: Agilent.
- Figure 1A: upon printing (and in the 100% view option of PDF) these figures are difficult to interpret. I suggest to make them larger by rearranging this figure (quite some white space left and right).

I have read this submission. I believe that I have an appropriate level of expertise to confirm that it is of an acceptable scientific standard, however I have significant reservations, as outlined above.

## References

[1] AgbulutOHuetANiederländerNPuceatMMenaschéPCoiraultC: Green fluorescent protein impairs actin-myosin interactions by binding to the actin-binding site of myosin. *J Biol Chem*.2007;282(14) : 10.1074/jbc.M610418200 10465-71 10.1074/jbc.M610418200 17289667

# Peer review: Reviewer response for version 1

**DOI:** 10.5256/f1000research.20214.r45625

**Published:** 2019-04-01

**Authors:** James R. Bamburg, Marcello Pignataro

**Affiliations:** 1 Department of Biochemistry and Molecular Biology, Colorado State University, Fort Collins, CO, USA

The specific aim of this Research Note is to demonstrate that *C. elegans* can be used as a useful *in vivo* model to study mutations in actin associated with human myopathies, specifically in this case, cardiomyopathies. The authors have thus expressed only in the muscle tissue of *C. elegans* GFP-chimeras of wild type (WT) actin and two actin mutants (E100K and P165A) equivalent to human actin mutants E99K and P164A both of which are known to cause hypertrophic cardiomyopathy in humans. The authors then study by fluorescence microscopy the localization of the actin-GFP in the body wall muscle, the sarcomeric organization using fluorescent phalloidin to visualize F-actin, and worm motility. Their main findings are that all of the GFP-actin proteins incorporate into the sarcomere with only the E100K mutant causing sarcomeric disarray and that both mutant actins, but not WT, form GFP-positive aggregates that do not stain with fluorescent phalloidin or with an array of actin antibodies even after implementing antigen retrieval protocols. From that, the authors suggest the relevance of *C. elegans* to study actin-related cardiomyopathies. However, actin aggregates have not been detected in mouse heart from animals expressing the E99K mutant nor have aggregates been reported in human cardiomyopathies from subjects with either of the mutations used here. Thus, we are left to wonder, as indeed the authors have themselves, if the aggregates are specific to nematode muscle, if aggregate formation by the mutant actins is enhanced by the presence of the GFP-tag, and what other molecules are found in the aggregates that might provide a clue to their composition. 

 As the authors have clearly written in their introduction, there is a need for further characterization of pathogenic mutations and expanding the available organisms to study specific diseases is always welcomed, as every model comes with their benefits and caveats. In spite of this, and the fact that the manuscript is well written and self-critical, there are some limitations in the studies presented that prevent us from approving its acceptance at this stage.

 Although it is understandable why the authors choose to express the fluorescently tagged version of the actin mutants, expression of the untagged versions should also have been examined to see if the sarcomere disruption by the E100K occurs in the absence of the GFP tag. It is recognized that under these conditions it would not be possible to obtain ratios of the expressed transgene with the endogenous protein, but with a fluorescent protein expressed from a different promoter, it should be possible to obtain worms with the untagged actin mutants to determine its effect on sarcomere disruption. As recognized by the authors, the GFP tag could increase the propensity of the construct to oligomerize/aggregate. Whether this could be driven by GFP aggregation on its own is not clear. It has been reported that GFP linked via its C-terminus to a 16 amino acid "degron" peptide is not effectively degraded but forms aggregates very similar to the ones observed here when expressed in the body wall muscle of *C. elegans* (Link *et al.*, 2006 ^1^). Since the aggregates observed in the current study have not been found to contain actin, one has to wonder if some cleavage has occurred to give rise to the same type of GFP-aggregates observed by Link *et al.* Why these do not form with the GFP-WT actin is unknown, but might reflect its greater stability to proteolysis. It would be useful to know if the number of aggregates correlate with total GFP intensity in a muscle cell.

 Also, the absolute level of the expressed proteins relative to endogenous actin is problematic. As recognized by the authors themselves, their western blot was made with whole organism lysate and the signal of endogenous actin was near saturation. Although the contribution of actin from regions outside the body wall muscle is thought by the author's to be low, if it were possible to isolate only the body wall muscle, the values would be more meaningful. For the results to mimic human disease pathology, the authors need to try and reproduce the ratio between mutant and endogenous actin. As the authors demonstrate decreased worm motility when overexpressing total WT GFP-actin, it is clear that the expression of exogenous actin must be kept low to avoid potential toxic effects, although in this case it is unclear if the decreased motility arises from the presence of the GFP-tag.

 While trying to explain the difference observed in their worm model with results from a previous paper (Song *et al.,* 2011) in which no aggregates were found with the E99K mutant, the first explanation given is “Thus, effects of these actin mutations appear to be dependent on cellular context.” Such an explanation actually negates the main purpose of the paper. We would thus recommend to first try to resolve the issues that have been identified in this review, because, as interesting as the starting idea is, there are some flaws that render the results difficult to interpret.

 Minor questions:

1. How do the authors explain the lack of correlation between sarcomeric disarray and worm motility?
2. Does the expression of any of the constructs lead to an increased mortality or to a reduced lifespan?
3. The nature of the aggregates that contain the GFP should be explored in more detail. Are they phase separated protein clusters such as one finds in stress granules and which can be identified by rapid diffusion within the structure if photobleached, or are these structured solid aggregates that might be able to be isolated, such as those from human from idiopathic dilated cardiomyopathy? The reason for their lack of actin staining needs to be identified since right now the title of the manuscript that states "human cardiomyopathy mutations, cause abnormal actin aggregation" cannot be used since no actin in the aggregates has been detected.

We have read this submission. We believe that we have an appropriate level of expertise to confirm that it is of an acceptable scientific standard, however we have significant reservations, as outlined above.

## References

[1] LinkCDFonteVHiesterBYergJFergusonJCsontosSSilvermanMASteinGH: Conversion of green fluorescent protein into a toxic, aggregation-prone protein by C-terminal addition of a short peptide. *J Biol Chem*.2006;281(3) : 10.1074/jbc.M505581200 1808-16 10.1074/jbc.M505581200 16239215

# Peer review: Reviewer response for version 1

**DOI:** 10.5256/f1000research.20214.r45621

**Published:** 2019-03-20

**Authors:** Amy Shaub Maddox

**Affiliations:** 1 Department of Biology, University of North Carolina at Chapel Hill, Chapel Hill, NC, USA

Several human muscle pathologies result from mutations in actin genes. To understand the cellular effects of such alleles, Hayashi and colleagues introduced the relevant mutations from human diseases into a muscle-enriched actin gene in *C. elegans*, an animal with a simple body plan and behavior. They found that mutant actins localize to muscle fibers but also form aggregates. At least at the low expression level examined, mutant actins did not cause animal movement defects.

 This is a clear manuscript with interesting potential. However, before it is suitable to be indexed, several points need to be addressed.

 Major point:

1. The authors should comment on the significant decrease in body bends exhibited by worms expressing GFP-ACT-4(WT). Could this relate to lower expression of this transgene – did this phenotype scale with expression level among the various isolates? Could it relate to the ability of the (lowly expressed) transgene to incorporate into muscle (measure GFP/F-actin staining ratio)? Arguably the relevant comparisons are between mutant and WT transgene, and there was a statistically significant difference in body bends for both these comparisons, though this was an increase. Are there known perturbations that lead to more beats per second than controls?

Minor points:

1. The qualitative assay for aggregate formation is unlikely to have the dynamic range necessary to discern among conditions of different severity. Doing so would probably require a quantitative assay such as measuring the range of fluorescence intensity throughout regions of the image, or the proportion of the image occupied by pixels over some intensity threshold.
2. The E100K mutation appears to have severely disrupted muscle fiber organization – the 9-11 prominent F-actin bundles apparent in WT cells are replaced with many more partially-overlapping wispy structures. This effect should be related to the disarray noted by Song *et al.*, 2011 (Discussion 3 ^rd ^sentence). The degree of subcellular morphological perturbation could be quantified with image analysis. It is interesting that despite this dramatic change, animal mobility is normal. 
3. It would be nice to note somewhere the origin of 3’ UTR (act-4).
4. “Turkey” should be “Tukey”; “Promotor” should be “promoter”.

I have read this submission. I believe that I have an appropriate level of expertise to confirm that it is of an acceptable scientific standard, however I have significant reservations, as outlined above.

# Peer review: Reviewer response for version 1

**DOI:** 10.5256/f1000research.20214.r45623

**Published:** 2019-03-20

**Authors:** Michael Glotzer

**Affiliations:** 1 Department of Molecular Genetics and Cell Biology, The University of Chicago, Chicago, IL, USA

This short note describes the ability of two cardiomyopathy associated actin alleles to incorporate into body wall myosin filaments in *C. elegans*. The authors find that whereas transgenic, tagged wild-type control actin ACT-4 incorporates well into the muscle, the two mutant variants do so with lower efficiency and also accumulate as non-filamentous aggregates. 

 This report is technically sound and the conclusions are appropriately drawn from the evidence provided. In future studies it would be of interest to incorporate these mutations into the genome so that the relative dosage of the mutant protein would better reflect that seen in cardiomyopathy patients.

I have read this submission. I believe that I have an appropriate level of expertise to confirm that it is of an acceptable scientific standard.
